# Biotechnological approaches for food frauds and rapid detection methods as preventive measures: a global perspective”, with a special emphasis on microfluidics

**DOI:** 10.1016/j.fochx.2026.104650

**Published:** 2026-10-21

**Authors:** Pratikshya Oli Chhetri, Muskan Kumari Gupta, Mradul Awasthi, Vinay Balodi, Ankita Hooda, S. Dhivya, Nikhil Pancholy, Himanshu Kumar Rai, Abhishek Dutt Tripathi, Aparna Agarwal

**Affiliations:** a Department of Dairy Science and Food Technology, Institute of Agricultural Sciences, Banaras Hindu University, Varanasi 22105, U.P., India; b Department of Food Technology, Lady Irwin College, University of Delhi, New Delhi, India

**Keywords:** Food fraud, Microfluidics, Blockchain-based traceability systems, Food authenticity

## Abstract

Food fraud is an enormous challenge in the world with impact on consumer health, stability of the market, and trust. This review focuses on the key types of food fraud, such as adulteration, substitution, mislabelling and counterfeiting, as well as their ramifications with respect to food safety and regulation. For food authentication, traditional analytical methods such as chromatography, spectroscopy, and DNA-based assays are reliable but can be complicated, require a large amount of analysis, and require centralized laboratory facilities. Microfluidic paper-based analytical devices (PADs) have thus become attractive platforms for rapid and decentralized food-fraud screening. Their application to milk and dairy products, honey, edible oil, meat, drinks, seafood and functional foods is discussed. They are discussed particularly with regard to their potential for miniaturized analysis, fast detection and integration into portable readout systems. Issues that still have to be addressed are sensitivity, cross-reactivity, matrix effects, standardization, and validation in various food matrices. The need for transparency and accountability in food supply chains may be further fostered by having integration with digital traceability technologies. In summary, the microfluidic platform is a complementary tool to existing analytical tools for rapid food authentication and food-fraud screening.

## Highlights

• Global food fraud threatens safety, markets, and consumer trust.
• Microfluidic PADs enable rapid, low-cost on-site detection of fraud.
• Future tools aim for higher sensitivity and stronger supply-chain transparency.

## 1 Introduction

Food fraud, adulteration, counterfeiting, and food criminality are concepts often seen in dialogues around global food safety. Entities such as the Global Food Safety Initiative (GFSI), the Grocery Manufacturers Association (GMA) in the United States, the US Pharmacopeia (USP), and European Parliament reports characterize food fraud as the deliberate and deceptive misrepresentation of food for monetary profit (Spink et al., 2019). Food fraud encompasses more than economically driven adulteration; it often involves deception, including counterfeiting, as well as adulteration, and may also include fraud, tampering, diversion, tax evasion, grey market operations, and overflow goods. Recent crises in the food business have intensified the problem of food fraud, amplifying the necessity to combat fraud across supply chains. Regrettably, existing food safety management methods are not explicitly tailored to address or alleviate fraud (Rizzuti, 2021).

Considering the deliberate nature of food fraud, strategies for addressing food safety must be reformed to prioritize fraud prevention and vulnerability mitigation, rather than only maintaining safety. Fraud vulnerability is a weakness or a flaw that enables negative events in a system. However, research exploring the antecedent factors that lead to the occurrence of food fraud vulnerability is scarce, whereas the existing data, therefore, draws its understanding fundamentally from other corporate crimes (Wang et al., 2024). Given the complexity of food fraud problems, it is necessary to examine the factors that spur fraudulent activities and the main factors that would make a food product more vulnerable.

Food fraud is still an issue related to food safety and authenticity in India, especially for products that are regularly consumed, including milk, spices, honey, and edible oil. The problem with the food supply chain is yet another reason why there is a need for a rapid and accurate method of authentication (Huisman & van Ruth, 2022).

Worldwide, modern technology is increasingly used for the sake of food authentication. As a way of dealing with food fraud, the food industry needs to shift from a safety lens approach to a risk-avoidance lens throughout the food supply chain (Manning et al., 2024).

### 1.1 The global scenario of food fraud

The incidence of food theft has increased tenfold over the previous four years, signifying a worldwide issue across many markets. This growth jeopardizes consumer safety and confidence while also presenting significant financial risks to businesses engaged in food manufacturing and delivery. Between 2020 and 2023, food fraud instances had a 1041% rise. This indicated a significant change in hazards faced by global food supply systems. Given that by May 2024, there were already 2479 registered occurrences, the figure may continue to rise; immediate action is required to address food fraud comprehensively (Dutfield & Suthersanen, 2022).

The increase, which has been reported by the SGS Digicomply Food Safety Intelligence Hub, underlines an increasing challenge to food safety professionals. It does not reflect improved detection and reporting but may show a gain in sophistication and frequency of fraudulent acts, pointing to what may affect consumer confidence and food safety. Adulteration, with 10,416 cases, dominates, reflecting widespread practices of unauthorized substance use to cut costs or alter quality. Process and logistics errors, documented in 3613 instances, illustrate vulnerabilities in the food supply chain's integrity. Lesser, but still significant, are issues like bio-contaminants and mislabeling, with over a thousand cases each, emphasizing the need for stringent control measures and accurate labeling practices. Beverages and meat products are also highly susceptible, with 2269 and 2246 incidents, respectively, while dairy, fruits, vegetables, and cereals add thousands more cases to the count, once again proving that the risk is across all food sectors (Rezazade et al., 2022) (Fig. 1.).

**Fig. 1:**
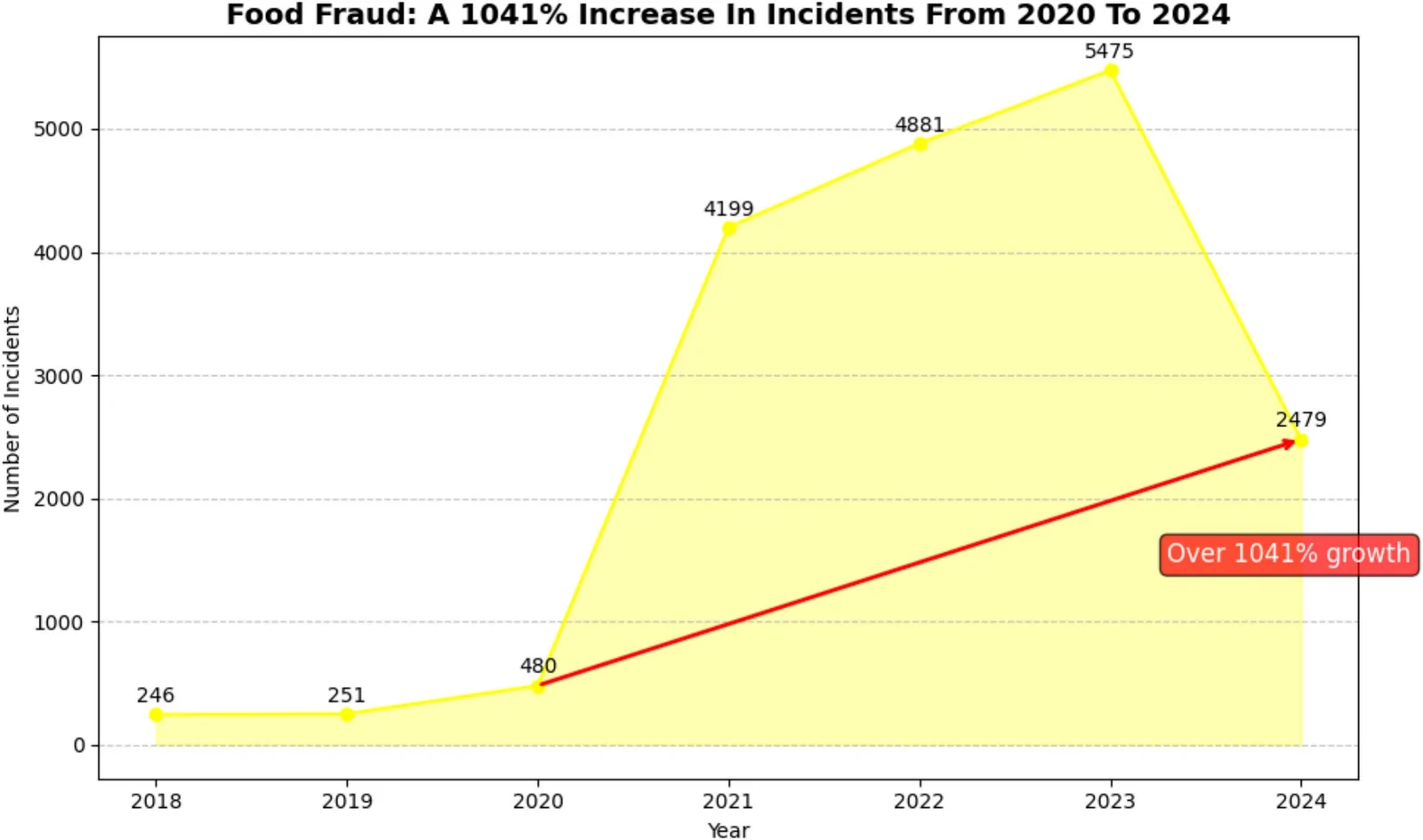
Increase in Food Fraud incidents from 2020 to 2024.

### 1.2 Motive behind food fraud

The primary reason for food fraud is financial gain, since offenders aim to profit by misrepresenting, adulterating, or counterfeiting food items. The pursuit of economic gain typically leads to substantial public health problems, since these fraudulent operations may introduce atypical and hazardous toxins. In contrast to conventional food safety risks that concentrate on recognized dangers such as germs, chemicals, and physical pollutants, food fraud often entails adulterants that are not usually scrutinized, exemplified by melamine, a plasticizer used to simulate protein in food quality assessments (Hellberg et al., 2020). It is essential to have prevention and authentication as well as fast detection strategies due to the above-mentioned economic reasons and vulnerabilities in the food industry.

## 2 Types of food fraud and commonly affected sectors

Food fraud results in revenue declines ranging from 13 to 80%, and stock values diminish between 37 and 75%. The price of an adulterated product was 1.5 to 4 times higher than that of an authentic product. Moreover, trust is influenced by food fraud. Trust in the industry dropped from 7% to 64%. The number of international firms participating in recalls varied from 5 to 22. The Global Food Safety Initiative asserts that the sector must endeavor to reduce the danger of food fraud (Table 1).

**Table 1:** Types of food fraud that are common.

Common types		Examples
Replacement	Dilution of a pure component with an adulterant or a combination of adulterants.	Manipulation to enhance the apparent protein content of milk Enhance the titratable acidity of lemon juice with citric acid. Excess moisture in frozen seafood. Omissions and fraudulent assertions
Addition	Incorporation of an adulterant to conceal an inferior component.	Utilization of color enhancers in substandard paprika.
Removal	Exclusion of a significant component without the consumers' awareness.	Extraction of nonpolar components (lipids and taste compounds) from paprika

### 2.1 Adulteration: definition, examples, and consequences

Food fraud and food adulteration are two major issues affecting the food landscape across the global food industry, most likely driven by economic benefits and weak oversight. Food fraud encompasses actions like imitation, dilution, substitution, or enshrinement of a product or service with a different or inferior quality one, deceptive labeling, or other practices intended mainly for profit-making. Food tampering, a type of food fraud, is the process of adding unfit or prohibited substances into foods; therefore, it is a potential danger to the health of consumers (Fig. 2.). Studies show that food fraud costs the food industry $10–$15 billion annually, or 10% of all food products. These include olive oil fraud, honey fraud, where high fructose syrup is mixed with honey, or other food products like juice, which is spiked with other compounds. Zhang, Ouyang, et al. (2020), Zhang, Ren, et al. (2020) and Zhang, Xu, et al. (2020) demonstrate that economically driven food fraud and adulteration constitute a developing and significant food safety issue in China. The study's findings may be used to assess food safety hazards and to prioritize areas for food policy development and implementation of food safety regulations. Adulteration of meat and fish products involves substituting species with lower economic value. The main objective is economic, but it may also have an effect on health. For instance, using soy, gluten, or milk proteins to substitute meat with low allergenic potential might result in meat products with high allergenic potential (Yang et al., 2022).

**Fig. 2:**
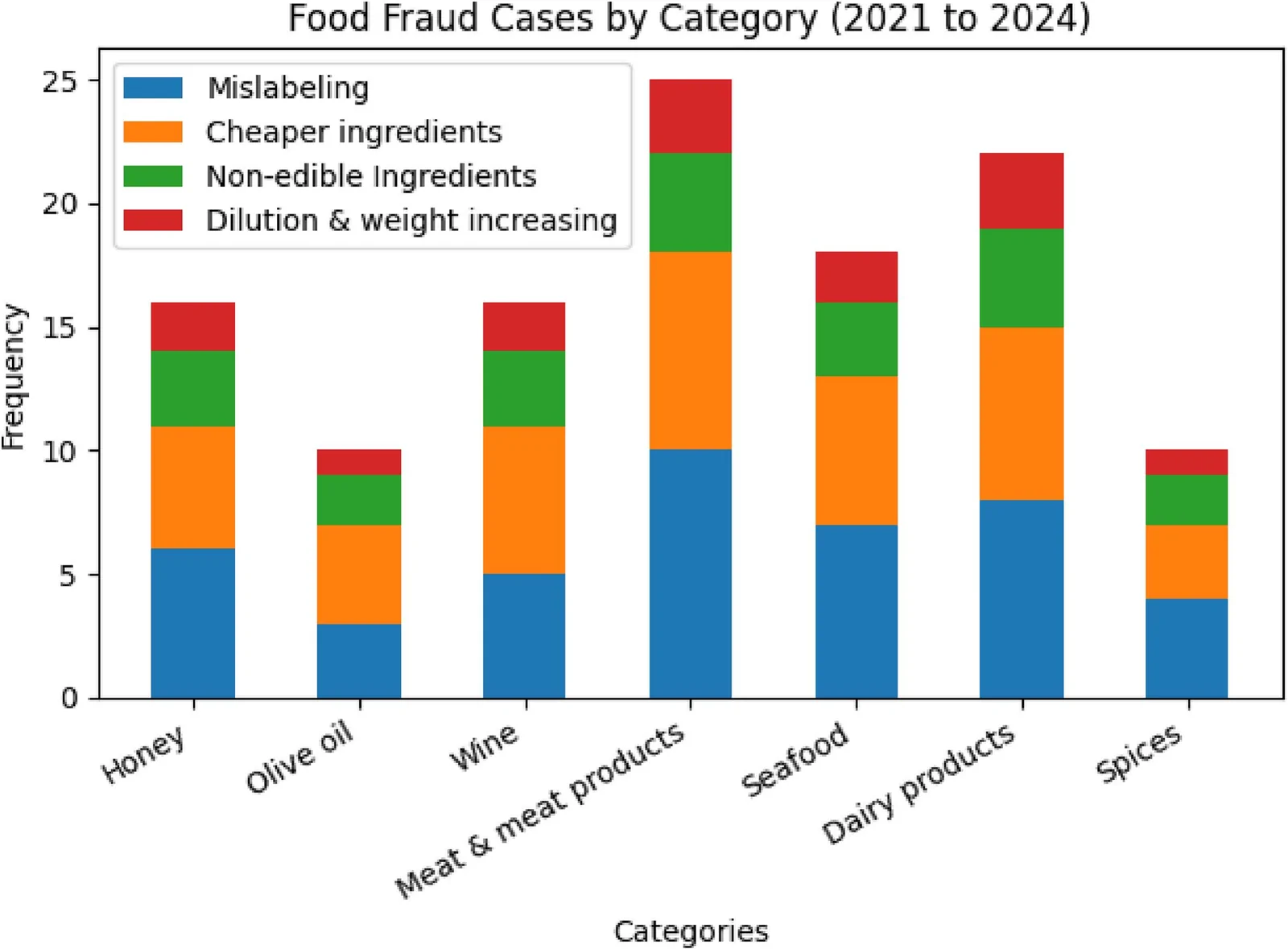
Food Fraud Cases by Category (2021–2024).

Dairy products may be adulterated by adding water (for example, to increase the amount of milk), starch, vegetable oils, and wheat flour to make the milk thicker and more viscous. Products such as formalin, calcium hydroxide, or sodium hydroxide are used to prolong the shelf life, while detergents are used to maintain the frothy appearance that is lost when milk is diluted with water. Urea is used to artificially increase nitrogen concentration and improve whiteness, creating an effect similar to that of melamine. The addition of lesser oils, such as vegetable oil, which usually don't have the same beneficial qualities as olive oil, contaminates olive oil. Sometimes, inexpensive wines and alcoholic beverages are promoted as high-end goods. Alcoholic beverages that may contain methanol are nonetheless very dangerous, even if mislabeling a cheap wine as more expensive does not pose a health risk to people (Fakhlaei et al., 2020).

### 2.2 Substitution and dilution: lower-quality ingredients masked as premium

Substitution and dilution are among the most common types of food fraud when inferior ingredients are misleadingly marketed as high-quality. Substitution entails the replacement of a costly item with a more economical alternative, sometimes without the consumer's awareness, such as exchanging olive oil for inferior vegetable oil or swapping premium fish species with less appealing ones. Dilution entails the amalgamation of premium goods with inferior or non-food components, such as the adulteration of milk, honey, or fruit juice, to augment volume and optimize earnings.

Enhancing consumer understanding, encouraging industry responsibility, and cultivating public-private partnerships are vital for establishing a robust food system. Although affluent nations have made considerable progress in combating food theft via legal and technical advancements, underdeveloped countries sometimes lack the requisite resources and infrastructure to handle these challenges efficiently. The use of rice, barley, or maize in coffee production, or substituting lower-quality coffee, are the main fraudulent activities. Spices sometimes include dangerous adulterants that endanger public health; for instance, they are frequently dyed with triphenylmethane, azo dyes, and lead chromate. Low-cost sweeteners such as high-fructose corn syrup, maize sugar syrup, inverted sugar syrup, cane sugar syrup, and rice syrup are often included in honey. Low-cost, inferior honeys might potentially be used as further alternatives.

### 2.3 Mislabeling and false claims

Food Fraud Vulnerabilities in the global food system have become common and critical within the global food chain when food items are intentionally compromised. Food fraud is a multifaceted and escalating concern within the global food supply chain, characterized by intentional misconduct. This includes unlawful activities such as misbranding, employing false trade descriptions, adulteration, impersonation, and deceptive claims regarding the origin, composition, preparation methods, and other attributes of food products. However, there is much greater agreement among all the stakeholders that food fraud is a major problem for consumers, businesses, governments, and regulatory agencies throughout the world and a threat to food safety, public health, consumer confidence, food supply chain integrity, and national economies. These include mislabeling and making presumably false claims, which are common types of food deception, the impacts of which can be widely felt in the food industry and by consumers.

Mislabeling means that false information on what the food product contains is provided, like incorrect descriptions on the contents of the pack, with meritless claims on the ingredients used, nutritional value, processing, or country of origin. The false claims also involve exaggerating or even inventing the benefits of the food product; for instance, labeling it as organic, gluten-free, or free of additives. A pervasive example of mislabeling is when companies substitute cheaper but substandard ingredients with expensive ones. For instance, fruit olive oil might be labeled as extra virgin when it is a blend of substandard oils; honey might be labeled 100% pure while it contains sugar syrup. These tricks are rather alarming in that they are hard to distinguish for the consumer, often needing some sort of laboratory testing to determine whether something is contaminated or mislabeled. Further, rare labelling mistakes and fake claims are not limited to any specific category of food – fresh and pre-processed fruits and vegetables, processed and packaged foods such as chips, beverages, and even nutritional supplements (Visciano & Schirone, 2021).

The European Union has put in place strict policies regarding the labeling of foods for the market to ensure that the product is not misleading the consumer and to create customer awareness of the content and origin of their food. The FDA is responsible for the implementation of specific labeling laws to prevent misleading ads or misrepresentation in the United States under the Food, Drug, and Cosmetic Act. However, food fraud persists despite implementing these rules due to the challenges of monitoring a global food supply chain and because it is highly lucrative. Firms might deceive customers through advertising products with health benefit claims, such as easy slimming, disease fighting, or performance enhancement, even when such claims lack evidence from scientific research. Signature foods that are marketed as cures for a number of conditions and often referred to as “super foods” or “miracle foods” for the most part have no scientific backing to support this type of claim. This is because deceptive health claims may also encourage the overuse of some of these items with compensating health effects (Soon & Wahab, 2021).

Combating fraud in the food industry, especially related to misrepresentation of products and making false claims, was identified as a need and a solution requiring multi-pronged action that includes building the robustness of regulation, enhancing food information to consumers and throughout the supply chain, and consumer awareness. Furthermore, increasing effectiveness in other functional areas of regulatory authorities that focus on the food supply chain and labeling regulations, or promoting cooperation between governments, industry corporations, and consumer groups, is important for addressing food fraud effectively. Some social marketing efforts might teach customers about the risks of mislabeling and misleading claims so that they can make better-informed purchase decisions.

### 2.4 Counterfeiting and brand imitation

Counterfeiting refers to the imitation of a particular food product, which includes its packaging, label, branding, or even the food content itself, with the intention to offer an unauthorized or substandard product to customers. Counterfeiting in the food industry involves faking higher-value-added products like olive oil, honey, and coffee through extension, replicating famous packaged products but using cheaper, adulterated ingredients. Most fake products come at cheaper prices, implying they are the real deal but contain hidden health dangers or are low-quality products. Pirated products are understood to be linked to organized crime, which obscures the identification and punishment of counterfeit products. Piracy involves producing foods that resemble the packaging, labeling, and branding of a reputable brand so that the customer is likely to succumb to the fake products, thus denying genuine brands a chance.

Rees (2020) report that experts at the Food Fraud Database believe that 10% of food in the marketplaces of wealthy countries is counterfeit, with some items regularly appearing on their blacklist, including chocolate, fish, pork, wine, olive oil, cheese, honey, saffron, and vodka.

## 3 Implications of food fraud on public health and safety

Food fraud poses significant implications for public health and safety, as it compromises the integrity of food products and can lead to severe health risks for consumers by exposing consumers to adulterated products, allergens, and even toxins. The human lust for profit has led to practices that disregard consumer safety, resulting in products that may cause mild to even life-threatening conditions (Kemsawasd et al., 2023). The increasing complexity of global food supply chains, coupled with inadequate regulatory oversight, exacerbates the vulnerability to fraudulent practices even further. Instances such as the introduction of harmful substances like melamine in milk or carcinogenic colorants in food products have proven to result in serious health issues (Spink, 2024). Other instances such as near-fatal poisoning from mislabeled seeds highlight the dangers of consuming adulterated products also such malpractices are difficult to trace and treat (Cao et al., 2023).

These problems pose an even greater threat to people in developing and undeveloped countries. Countries with very few resources and low-income populations, such as many regions, particularly in Africa, lack stringent food safety regulations; most developed countries try dumping their substandard food products into these countries, putting them at an increased risk of food fraud (Abia et al., 2024). In the African country of Ghana, several instances of adulteration with substances like sawdust and carbide have become prevalent, necessitating urgent action to combat these threats effectively (Sulley et al., 2023). Bichi and Hamza (2024) delved into the condition of food contamination and reported that among samples from Bichi Central Market, Kano State, Nigeria, 90% of street food samples showed bacterial contamination with the presence of harmful pathogens like *Salmonella* and *E. coli*. Whereas Countries like the USA, the UK, Canada, and China have been relevant in combating food fraud (Bannor et al., 2023). Even among consumers, children and adolescents are at an even higher risk. According to Everstine et al. (2024), 15,575 food fraud instances have been recorded from 1980 to 2022, among which milk, olive oil, and honey comprised the highest-risk ingredients.

### 3.1 Allergic reactions and foodborne illnesses

Allergic reactions and foodborne illnesses are two significant health concerns that often present overlapping symptoms, complicating diagnosis and management. Both conditions can arise from the consumption of similar foods, such as milk, eggs, fish, and shellfish, leading to confusion in clinical settings. Even the symptoms of both food allergies and foodborne illnesses can manifest as nausea, vomiting, diarrhea, and abdominal pain (Koire, 2024). The onset time of symptoms for both cases is also similar and can occur rapidly after ingestion of the food (Patel et al., 2022). Hence, understanding the distinctions between these two issues is crucial for effective treatment and prevention strategies.

#### 3.1.1 Food allergies

Food allergies are an autoimmune response triggered by specific proteins perceived as harmful, with symptoms ranging from mild to severe, including anaphylaxis (Morya et al., 2022). Some of the major allergens and their triggers have been mentioned in Table 2. These allergies are mediated by several antibodies, with IgE being the main cause and IgA and IgG suppressing allergic reactions (Udoye et al., 2023). Worm et al. (2017) have highlighted that food substances can act as triggers for severe allergic reactions among which 271 anaphylactic reactions from food recorded with children experiencing the most vulnerable group. Although several foods have been identified as triggers, the most common ones include peanuts, tree nuts, and cow's milk, with specific demographics at higher risk, such as children and individuals with asthma (Novembre et al., 2024). According to recent data, the incidence of food-induced anaphylaxis hospitalizations was recorded at 0.77 cases per 100,000 person-years in Spain, with children under 15 years experiencing the highest rate of 3.68 (Jiménez-García et al., 2024). In Ireland, Crealey and Byrne (2023) have reported that the annual incidence of accidental allergic reactions (AARs) was 4.5% in schools and 5% in preschool childcare settings; what's more concerning was that 22% of AARs in schools resulted in anaphylaxis, with no adrenaline administered by staff during these incidents. Whereas in the UK, although a rise in food-induced anaphylaxis hospitalizations has been reported from 1.23 to 4.02 per 100,000 population from 1998 to 2018, a decrease in the case fatality rate has been observed (Turner et al., 2024). With ongoing research and consumer awareness, more and more people are becoming aware of food allergies, which has resulted in timely responses decreasing fatality rates.

**Table 2:** Major food allergens with their respective trigger compounds.

S. No.	Food Allergen	Trigger compound	Reference
1	Cow's Milk	β-Lactoglobulin	Chruszcz et al., 2021
2	Peanuts	Ara h 8	Jappe et al., 2019
3	Wheat	Gluten	Barros & Cosme, 2013
4	Eggs	Ovomucoid	Matsuo et al., 2015
5	Shellfish	Tropomyosin	Matsuo et al., 2015
6	Soy	Gly m 4	Masilamani et al., 2012
7	Tree Nuts	Various Proteins	Barros & Cosme, 2013

#### 3.1.2 Foodborne illness

Whereas the term foodborne illness relates to pathogens or toxins in contaminated food, leading to infections or intoxications (Koire, 2024) (Fig. 3), the major foodborne illnesses have been summarized in Table 3. These are mainly caused by a variety of pathogens, including bacteria, viruses, and parasites. Unlike food allergies, foodborne illnesses influence every person however children are more vulnerable due to a weaker and developing immune system, also developing and underdeveloped reasons are at a higher risk of food borne illnesses with African and Southeast Asian regions experiencing the highest per population risk. These illnesses have caused over one million deaths annually, with diarrheal diseases and invasive infections due to non-typhoidal *Salmonella enterica* being most responsible (Kirk et al., 2015). The World Health Organization has estimated 33 million disability-adjusted life years (DALYs) lost in 2010 to foodborne illnesses, with a significant portion affecting children under five years old (Cissé, 2019). With the recent researches and improving food safety systems the issue of these illnesses has been controlled for some extend but still challenges such as antimicrobial resistance and bacterial, viral, and parasitic pathogens evolving and emerging are making control complex (Haydar et al., 2019).

**Fig. 3:**
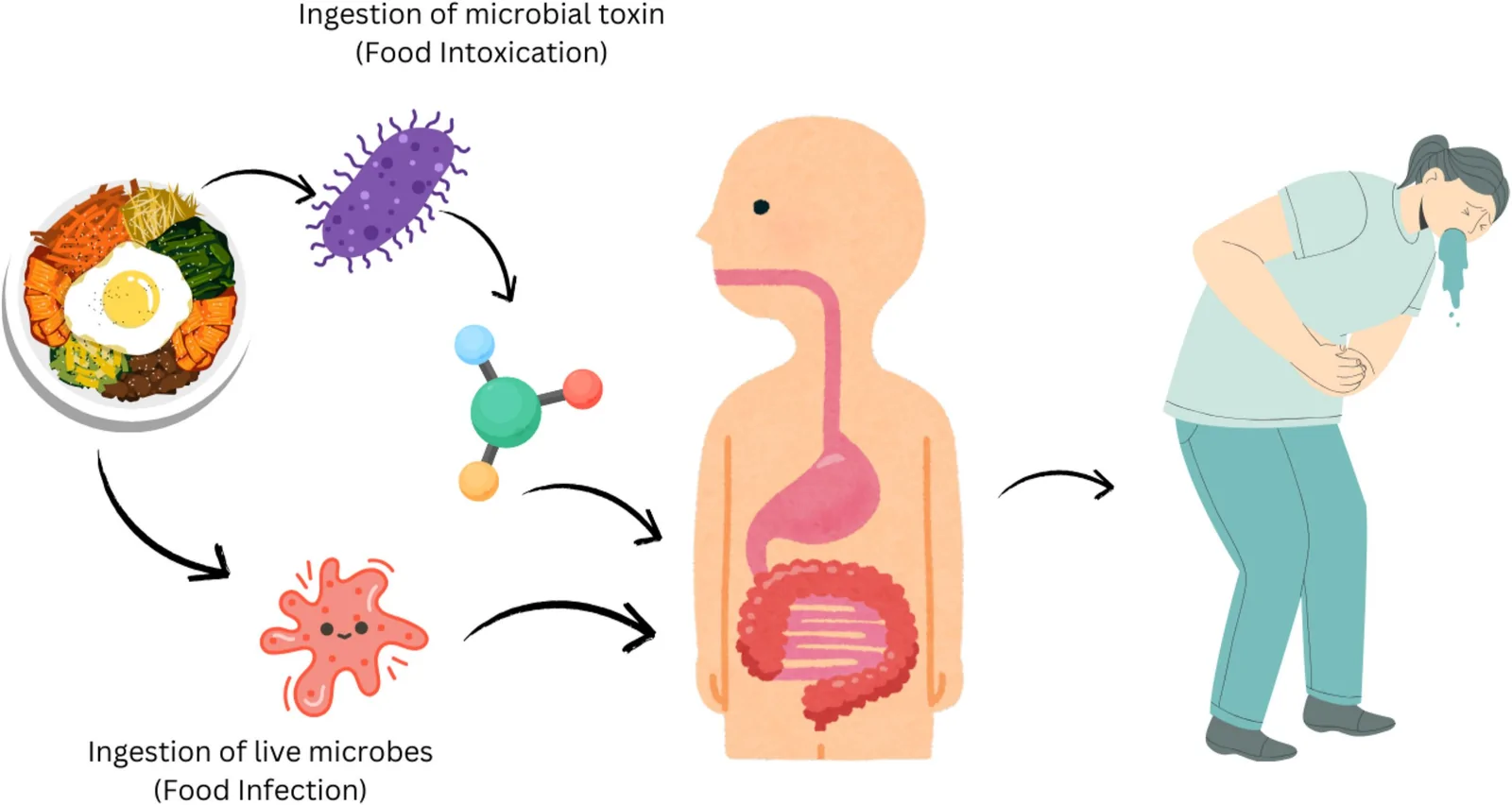
Food borne illnesses.

**Table 3:** Major food borne illnesses with there causative agents.

Disease	Causative Agent	Symptoms	Onset Time	Fatality Rate	References
Norovirus	Norovirus	Diarrhea, vomiting, nausea, stomach pain	12–48 h	Low, but high incidence	(Kirk et al., 2015)
Campylobacteriosis	Campylobacter spp.	Diarrhea, cramping, abdominal pain, fever	2–5 days	Low, but common	(Havelaar et al., 2015)
Salmonellosis	Salmonella spp.	Diarrhea, fever, abdominal cramps	6–72 h	0.5%	(Mead et al., 1999; (Behravesh et al., 2011)
Listeriosis	*Listeria monocytogenes*	Fever, muscle aches, nausea, diarrhea; severe cases can lead to meningitis	1–4 weeks	16.9%	(Behravesh et al., 2011)
Toxoplasmosis	Toxoplasma gondii	Flu-like symptoms; severe cases can affect the brain, eyes, or other organs	1–3 weeks	High in immunocompromised	(Torgerson et al., 2015)
Yersiniosis	*Yersinia enterocolitica*	Fever, abdominal pain, diarrhea	4–7 days	Low	(Chlebicz & Śliżewska, 2018)
Shigellosis	Shigella spp.	Diarrhea, fever, stomach cramps	1–2 days	0.1%	(Behravesh et al., 2011)
Vibriosis	Vibrio spp.	Diarrhea, abdominal cramping, nausea, vomiting	24 h	5.8%	(Behravesh et al., 2011)

### 3.2 Toxicity from unapproved additives and adulterants

Every chemical has an effect on the human body, and chemicals used in the food industry as additives must be safe for consumption, but often it has been observed that food manufacturers use unapproved additives to reduce costs or to achieve some other benefit in their product. Most times, unapproved additives are added as adulterants to hide some undesirable property of these food products, examples being talc powder in supplements, silica salts in powders, and many more. The non-uniform global standard for the safety assessment of food additives further leads to potential gaps in consumer protection (Kumar et al., 2019). Even many additives, particularly those used in veterinary drugs and pesticides, have not undergone rigorous toxicity evaluations; these products are often not tested in the products due to these low chances and lack of testing methods, resulting in residues in food products (Klátyik et al., 2017). Nagy et al. (2019) have also suggested that current testing requirements for pesticide product formulations are inadequate, as they mainly focus on individual ingredients without considering the interactions between them, which may have implications for human health if consumed.

Most artificial additives, such as colorants, preservatives, and sweeteners, have been linked to mental health disorders, cardiovascular diseases, metabolic syndrome, and potential carcinogenic effects. Food colors like azo dye food colorants and preservatives like sodium benzoate have been associated with behavioral disturbances in children and neurotoxic properties in animal models (Warner, 2024). One major incident of such a case was observed with the use of MSG (Monosodium Glutamate), a common food additive that is extensively used in Chinese foods worldwide, which has now been linked to hypertension, obesity, gastrointestinal issues, and neurological impairments, although its effects are dosage- and exposure-duration-dependent (Chakraborty, 2024). Türkmen (2014) hasalso suggested that adulterants in illegal substances, such as paracetamol, caffeine, griseofulvin, and phenacetin, can have more harmful toxic effects and endanger the lives of users. Various toxic adulterants such as lidocaine, caffeine, and diphenhydramine have been detected in umbilical cord tissue samples, which can result in potential in utero exposure to drug toxicity (Nelson et al., 2021).

Another major food category, the nutraceutical, is facing the heat of several intentional additives. From 2007 to 2016, 776 dietary supplements have been reported to contain unapproved pharmaceutical ingredients, with sildenafil, sibutramine, and synthetic steroids being the most common adulterants (Tucker et al., 2018). Various other unapproved additives, like surfactants, exhibit additive, synergistic, or antagonistic effects, necessitating thorough toxicological evaluation for environmental risk assessment (Klátyik et al., 2017). These problems of adulterants are further intensified in developing and underdeveloped countries where limited resources make the testing more difficult. In packet milk samples from Aurangabad, India, various harmful additives and adulterants such as starch, boric acid, and formalin have been reported which can pose health risks and toxicity to consumers (Mirza et al., 2018).

### 3.3 Long-term health risks from misidentified ingredients

Intentional adulteration containing harmful chemicals has resulted in numerous illnesses and deaths, where most cases are driven by economic gain, leading to widespread public health risks; these cases highlight the severe consequences of misidentified ingredients (Brevett & Cox, 2024). High levels of counterfeiting in food products can lead to unintentional allergen exposure, resulting in severe allergic reactions; in one instance, undeclared allergens in canned meats have been reported, which poses serious health risks, especially for individuals with allergies (Soldatova & Guseva, 2022). Misidentified ingredients can lead to severe allergic reactions, including anaphylaxis, particularly from hidden allergens like peanuts and soy; the long-term health risks from these products include chronic allergic responses and potentially fatal outcomes from accidental consumption (Malmheden 2014). According to research by Choudhary et al. (2024) in India, 15–20% of spices are reported to be adulterated, which can cause gastrointestinal issues and long-term health complications due to the presence of harmful additives.

Many consumer products have been found to contain chemicals such as phthalates, bisphenol A, and polybrominated diphenyl ethers, which may either be added or may have migrated from food contact materials, personal care items, and electronics, and can lead to toxic effects if not identified (Li & Suh, 2019). Some ingredients sold as flavors have various compounds that are not labeled but may be harmful, as found in flavored e-cigarettes; particularly with cherry flavor contain benzaldehyde, which can irritate respiratory airways also long-term inhalation of such flavoring ingredients poses potential health risks (Kośmider et al., 2016). Histological analysis of food products has revealed many plant-based proteins and thickening agents, which could trigger allergic reactions in sensitive individuals; if not mentioned, they can go unnoticed and end up in consumers' foods (Soldatova & Guseva, 2022). Many times, manufacturers use synthetic colors to downplay the changes during processing or enhance the appearance of food products, but long-term consumption of foods with artificial additives, particularly artificial colorants and preservatives, may increase the risk of mental health disorders, cardiovascular diseases, and metabolic syndrome, even studies have shown a correlation between high intake of emulsifiers and cardiovascular diseases in long-term consumption (Warner, 2024).

## 4 Traditional and emerging technologies for food fraud detection

The detection of food fraud includes several types of analyses that are based on chemistry, chromatography, molecular biology, spectrometry, or even protein techniques. However, despite being highly accurate and sensitive, most of these techniques need sophisticated equipment, skilled specialists, sample preparation, or laboratory settings (Hassoun et al., 2020). Hence, new technologies have been developed in the past few years with the goal of overcoming traditional methodology pitfalls and enhancing food deceit detection. Spectroscopic techniques such as vibrational spectroscopy, nuclear magnetic resonance (NMR) spectroscopy, and fluorescence spectroscopy have gained growing significance due to being quick, non-destructive, and able to provide detailed evaluations of food authenticity. These methods, often followed by chemometric analysis, hold much potential in establishing fraudulence in foods of animal origin and other food categories (Chaudhary et al. (2022); Hassoun et al., 2020).

### 4.1 Chromatography and spectroscopy techniques

To this day, thin-layer chromatography (TLC) remains an important and prevalent technique for evaluating food authenticity and adulterant detection in foods. It tends to be used in conjunction with more sophisticated techniques such as LC-MS/MS that are needed for analyzing intricate food matrices like oils, spices, and beverages (Sherma & Rabel, 2018). The merging of chromatographic methods with chemometric tools has also made the techniques more robust since this convergence provides a more thorough characterization of foodstuffs, in terms of food composition and authenticity through analysis of targeted biomolecules (Karabagias, 2020).

In the context of spectroscopy, newer techniques, such as vibrational spectroscopy, nuclear magnetic resonance (NMR), and fluorescence spectroscopy, have also been established that provide quick, non-destructive analysis and are being increasingly used to identify authenticity problems in food items. These innovations have centered on non-targeted spectroscopy methods as a contrast to conventional targeted procedures for food fraud analysis (Hassoun et al. (2020); McGrath et al. (2018)). In Molecular spectroscopic methods, several technologies such as Near Infrared Spectroscopy (NIRS), Infrared Spectroscopy (IR), and Hyperspectral Imaging (HSI) have emerged as important tools because they are inexpensive, require little sample preparation, and can offer extensive compositional analysis, especially in determining geographical origin and detecting adulteration (Zaukuu et al., 2022). Raman spectroscopy methods, including advanced Surface-Enhanced Raman Spectroscopy (SERS) methods, are also being praised for their rapid and non-invasive capabilities in detecting muscle food adulteration; further integration with chemometrics has enhanced their practical application in identifying food fraud (Lin & He, 2019).

### 4.2 DNA-based techniques for species and ingredient authentication

DNA-based techniques have garnered a lot of attention in recent times due to their high sensitivity and specificity and have become essential tools for authenticating species and ingredients in food products, addressing very concerning issues like food fraud, mislabeling, and consumer protection. Polymerase Chain Reaction (PCR) and its advancements such as Real-time PCR, multiplex PCR, and PCR-RFLP have proved to be useful in species and breed differentiation in food products; these are particularly favored for their high sensitivity and ability to detect adulterants even in trace amounts (Lo and Shaw (2018); Baptista et al. (2021)). Techniques like Next-Generation Sequencing (NGS) can provide high-throughput species identification even allowing for comprehensive analysis of many food products, this technique has been particularly useful for detecting unsearched ingredients and complex mixtures of food products with high precision (Lo & Shaw, 2018). NGS technologies, such as Illumina MiSeq, have been reported to enable precise species identification in meat products by amplifying mitochondrial DNA regions (He et al., 2024).

Apart from these traditional processes, rapid methods like DNA Biosensors have been developed; for example, paper-based DNA biosensors have been developed for testing food authenticity, offering high detectability and specificity in detecting adulteration even up to 0.01%, which is 10 times higher than most other known methods being used. These biosensors are particularly effective for dairy products and are being developed for most other food types (Bougadi & Kalogianni, 2020). DNA biochips like the LCD (Density Chip DNA) Array kit used in food forensics have shown high specificity and sensitivity, making them a useful tool for detecting common adulterants, particularly in animal-derived foods (Beltramo et al. (2017); Baptista et al. (2021)).

### 4.3 Sensory evaluation and organoleptic analysis

Sensory and Organoleptic evaluation of foods mainly includes the use of the senses of sight, sound, taste, smell, and touch; it is crucial for ensuring organoleptic quality and ensuring the right ingredients, recipes, and processes are in place in any food manufacturing facility. This method of food testing has been used historically for analysis of food; although cost-effective, iton the personal experience of expert assessors, lacks reproducibility. However, methods have now been improved, as more structured methods using panels of assessors and statistical techniques have been developed to improve reliability and reproducibility (Piana et al., 2004); descriptor methods and sensory vocabulary have been developed, which are crucial for standardizing evaluations. Organoleptic analysis particularly evaluates the sensory attributes such as taste, aroma, and texture of food products. These methods are essential for products like cheese and tomatoes, where complex profiles are formed during processing (Causse et al., 2001). Among sensory evaluation methods, a novel approach, neuroorganoleptic analysis, integrates traditional sensory testing with psychophysiological data to assess consumer preferences more accurately. In this method, apart from the consumer responses, the physiological responses, like heart rate variability, are correlated with self-reported preferences, which offers a much deeper understanding of consumer behavior (Valente et al., 2021).

In recent times, the technology has advances we have started to develop instrumental methods which could mimic the human behavior and senses. Non-invasive and rapid methods such as hyperspectral imaging (HSI) allow for the assessment of sensory properties like color, texture, and freshness without destroying the sample; combined with multivariate analysis, HSI offers a promising approach for real-time monitoring of food flavor in industrial applications (Özdoğan et al., 2021). In the flavor industry, metabolomics has emerged as a valuable tool; it provides valuable information on the chemical composition of food products, which can be related to sensory properties (Shu et al., 2022). Many electronic replicas of human sensory organs have been created, such as *E*-nose and E-tongue, which can act as a powerful and objective inspection tool for food quality assessment, even outperforming individual techniques in various applications (Kiani et al., 2016). Among these, E-nose has shown promising results as a non-destructive alternative for quality inspection of agricultural and food products, ensuring maximum consumer preference and safety (Ali et al., 2020), while also providing a wider scope for testing of products; with E-nose and E-tongue, even certain compounds can be tested for flavor that could not be tested by human subjects like allergens, untested ingredients, or new compounds with no toxicological data available.

### 4.4 Limitations of conventional detection methods

The traditional method of food testing, although used for a long time and evolved with technological advancements, is still facing a lot of heat from the rising demand for food safety in recent times. Most chromatographic techniques, such as high-performance liquid chromatography (HPLC) and gas chromatography (GC), require vary sophisticated equipment's with trained personnel's and often require complex sample preparation and operation, which can be expensive and time-consuming for the food manufacturers (He, Bai, et al., 2020b, 2020c; He, Joseph, et al., 2020). Some compounds in food products have poor electrochemical activity, which necessitates additional precolumn derivatization so as to make them detectable, which further complicates the problem (Cuadros-Rodríguez et al., 2021). One major limitation also comes with the standardization of methods, as most food testing operations lack official standardized chromatographic protocols, which limit the scope of chromatographic fingerprinting testing (Sontag et al., 2019).

DNA-based food adulteration and authentication techniques have garnered a lot of attention for their highly specific and accurate results, yet most of these methods are yet to be improved to scale up. Among the many challenges in DNA-based food authentication, degradation of DNA and the presence of inhibitors affecting PCR amplification have been the most concerning one necessitates the need for more robust extraction and amplification protocols (Lo & Shaw, 2018). Higher cost and the need for sophisticated laboratory setups are the other problems that affect the widespread use of these methods (Lockley & Bardsley, 2000).

Although newer methods in Sensory evaluation, like using trained panels and controlled protocols, have overcome the problems of reproducibility, the training costs and overhead costs of hiring a trained assessor make the process complex and costly, which often limits their regular use. Most of the times, the assessors are trained with specialty in one product like wine, tea or apples which makes then highly specific for the industry and lesser known industries have to spend a large sum for finding a suitable person. Also in industries where the sample size is quite large, like in olive oil and agricultural products, there is need for a large number of assessors as most human would get saturated after evaluation of a limited number of products which necessitates the requirement of instrumental analysis to cope up with large volumes of samples (Quintanilla-Casas et al., 2020). To overcome these limitations, devices like e-nose and e-tongue have been developed and improved in sensitivity and selectivity, yet they are still not accurate for complex mixtures or low-concentration compounds, which potentially leads to less accurate results. Unlike the human assessors, the accuracy of these devices is heavily dependent on strict control over sample preparation and data processing, but any deviation in these steps can lead to inconsistent results (Tan & Xu, 2020). Although these methods can prove to be affordable in long terms yet their initial setup costs and maintenances are expensive which limit their use to large institutions only. The complexity of integrating and calibrating the sensors and software for these devices further adds to the complexity for small industries with not much of a highly skilled workforce.

### 4.5 Microfluidics, rapid testing for on-site, portable testing (advantages and limitations)

Microfluidics is a technique used in miniature analysis systems that allow sample manipulation and detection within small-volume systems. Therefore, microfluidics is highly relevant for fast and field analysis of foods.

Most conventional channel-based microfluidic devices have low productivity and high labor costs, which limit their scalability in the food industry; also, these devices are prone to clogging, which can significantly reduce their efficiency (He, Bai, et al., 2020b, 2020c; He, Joseph, et al., 2020). The major advantage of microfluidic devices is their integration of various processes, but in complex food matrices, the presence of various different components in the same sample can cause difficulty in the detection process, affecting the accuracy of pathogen detection (Kant et al., 2018). With such complex integrated processes, there is a requirement for highly skilled personnel to operate such devices, which limits their widespread use in the food industry.

### 4.6 Artificial intelligence (AI) and machine learning (ML) in food fraud detection

Artificial Intelligence (AI) and Machine Learning (ML) utilize sophisticated algorithms and data analysis methods for improving the speed, accuracy, and efficiency of detecting fraudulent activities in foods. With the integration of these sophisticated technologies, faster detection of adulteration and authenticity problems could be achieved through predictive analysis. Additionally, the integration of several methods, such as the union of machine vision with deep learning technology, enables non-destructive and real-time examination of food quality, which could be harnessed to improve detection potential (Shen et al., 2024). These technologies are being used in quality control, inspection, and supply chain, improving efficiency and traceability, which could go a long way to improve consumer confidence (Gbashi & Njobeh, 2024).

These technologies have also been found to be effective in analyzing complex process outcomes, such as in flavor business, where AI has been utilized to examine complex and distinctive aroma profiles using integrated electronic nose technology and chemometric techniques (Naeem, 2024). Explainable AI methods (XAI) can be used to translate the predictions of machine learning models, making them effective in food fraud detection. In this type of case study, a food fraud dataset was constructed wherein XAI tools revealed the possibility of interpreting deep learning models; this research illustrated the advantages and disadvantages of such systems in testing food adulteration (Buyuktepe et al., 2023).

With advancements in semiconductors and technology, AI is becoming more accessible for the food industry and is being used in various places for full automation of plants, still these technologies are yet to be fully integrated in the food fraud detection. Various challenges have limited the use of AI and ML including the concern for data privacy concerns, as most AI are based on algorithms any potential biases can result in incorrect predictions (Shen et al., 2024). With time, the complexity of supply chains is increasing, and fraudsters have been evolving their methods, which requires continuous updating of databases and AI models to tackle these issues (Gbashi & Njobeh, 2024).

### 4.7 Blockchain and digital traceability in supply chain transparency

Blockchain technology is an emerging method for recording information that offers a more decentralized way; this technology has been useful for enhancing transparency in supply chains by backtracking and tracing products from origin to consumer, ensuring the authenticity and integrity of supply chain data. With this technology, shortcomings of centralized traceability systems have been addressed, which has enhanced visibility in various distribution network designs. This transparency has been particularly used in fields like food and pharmaceuticals where safety and security are of utmost importance (Sunny et al., 2020).

With recent advancements, blockchain technology has been integrated with other technologies like the Internet of Things (IoT) and artificial intelligence (AI), further enhancing its capabilities for supply chain traceability (Fig. 4). With IoT technology, devices have been created that can provide real-time data on product conditions and locations; this data, coupled with blockchain, could be used for transparent data sharing across the supply chain, thus ensuring the safety of products from farm to fork (Wong et al., 2024). This has further been integrated with AI systems to analyze this data and report any fluctuations that could pose a threat to product safety, improving decision-making and operational efficiency (Khan et al., 2022). The food industry has been using blockchain to achieve sustainability goals of reducing environmental impact by ensuring traceability of products (Chandan, John and Potdar, 2023). When combined with Industry 4.0 and Web 3.0, there has been much improves food traceability and traceability of various other supply-chain operations which has provided an overall system for better management, automation, and sustainability (Ellahi et al., 2023).

**Fig. 4:**
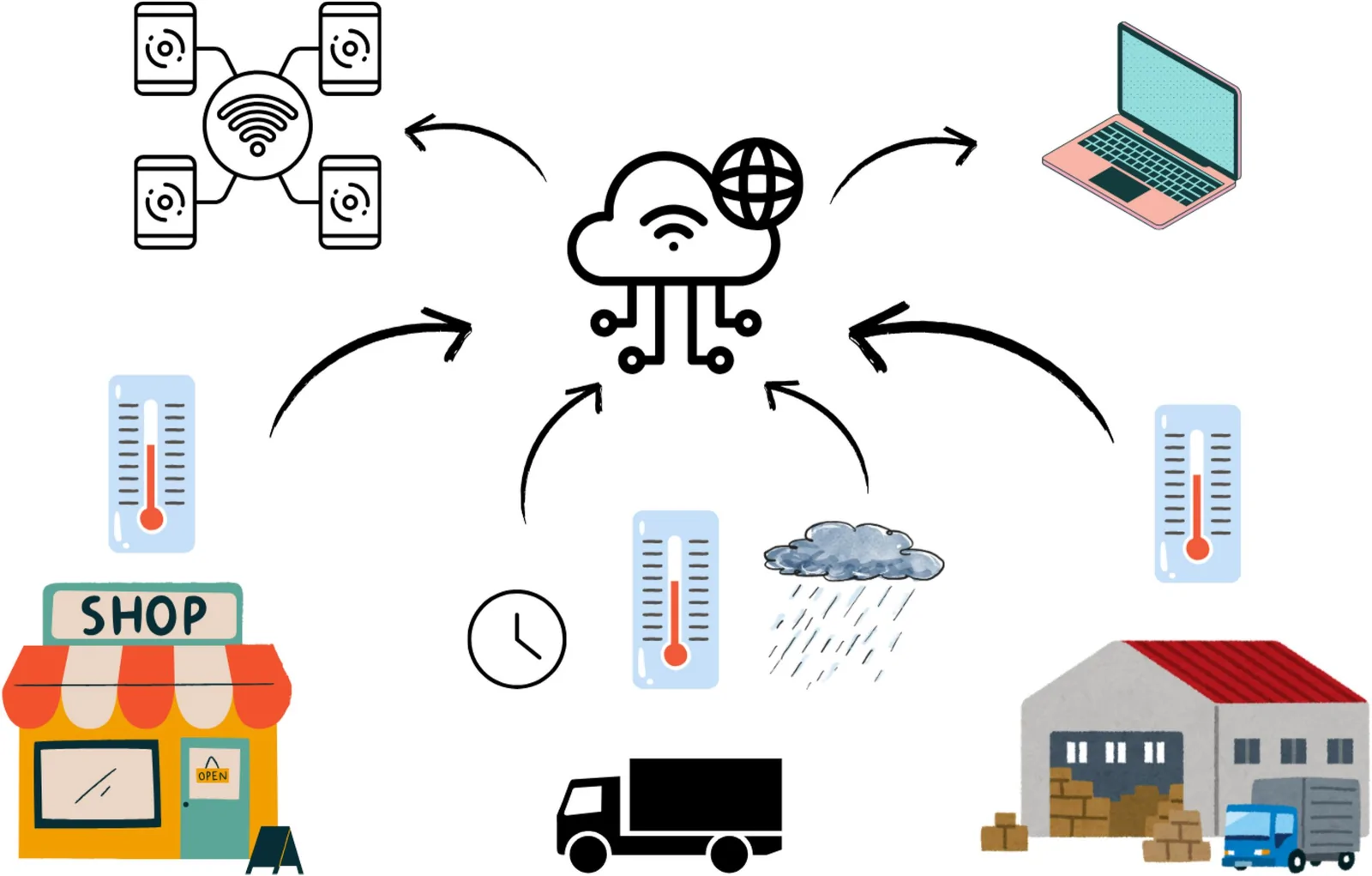
Internet of Things for quality inspection of food products at every point of supply chain.

Despite the huge amount of benefits being provided by blockchain technology, there are still various challenges such as high implementation costs, scalability, and regulatory compliance that have hhinderedthe widespread adoption of blockchain technology in the food supply chains (Baranidharan et al., 2025). While blockchain technology improves transparency and traceability, it works on the retention and analysis of data related to the supply chains; as product records accumulate, it increases the total data storage cost and decreases the efficiency of the system. Another major barrier is he total cost of implementation, as there is minimal human interference; most of these blockchain systems use sensors for data collection, which requires a high initial setup cost and additional maintenance costs over time, which limits their implementation for some organizations (Dasaklis et al., 2022).

Overall, conventional analytical techniques provide high sensitivity and reliable identification of food fraud, but their dependence on sophisticated instrumentation, skilled personnel, and laboratory-based workflows can limit their use for rapid screening and on-site applications. From the studies reviewed, this creates a clear need for complementary approaches that can retain adequate analytical performance while reducing analysis time, sample-processing requirements, and operational complexity. Microfluidic technologies are particularly relevant in this context because they can integrate several analytical steps within miniaturized platforms.

## 5 Microfluidic technology for food fraud detection

Microfluidic technology is an advanced technique that allows handling and analyzing very small amounts of liquid inside miniaturized systems. Combining this technology with different detection methods like colorimetric, electrochemical, nucleic-acid-based, immunoassay, and optical detection methods has opened up new possibilities in the field of rapid food authentication and adulterant testing. μPADs (microfluidic paper-based analytical devices) are especially useful as paper-based media are capable of performing capillary-driven fluid movement and thus allow portable and field-based analysis (Dou et al., 2015; Yehia et al., 2020).

Fabrication of microfluidic devices is performed using several techniques like soft lithography, molding, laser cutting, and 3D printing depending on the requirements of the system design (Alahmad et al., 2024; Yehia et al., 2020).

From the literature reviewed, one of the major benefits of microfluidics in the context of food fraud detection is not only miniaturization but the potential for integration of sampling, reactions, and detection in one system. Integration has the potential to cut down the analysis time and use of reagents, and even facilitate decentralized testing. However, miniaturization is not an absolute criterion for successful detection, and one must consider performance characteristics along with matrix complexity, specificity, reproducibility, and ease of use.

## 6 Microfluidic technology for food fraud detection

Microfluidic technology refers to manipulation and analysis of small fluid volumes in a miniaturized platform. Integration of microfluidics with various detection technologies such as colorimetry, electrochemistry, nucleic acids, immunoassays, and optics has made possible rapid food testing and adulterant detection. μPADs are especially promising because paper substrates allow for capillary-mediated flow of liquids and miniaturization of assays (Dou et al., 2015; Yehia et al., 2020).

Microfluidic devices can be constructed by methods such as soft lithography, molding, laser ablation, and 3D printing, depending on the specific device design and application (Alahmad et al., 2024; Yehia et al., 2020).

According to the literature overview, one of the advantages of using microfluidics in food fraud detection is not only its miniaturized nature but also its ability to combine sampling, reactions, and detection in one system. This could result in reduced reaction times and reagents needed for the process, as well as the ability to perform decentralized testing. Nevertheless, miniaturization does not automatically provide for successful detection, and analytical characteristics should also be considered.

### 6.1 Principles of microfluidics in food testing

#### 6.1.1 Dimensionless numbers

As shown by the governing equation of the liquid-liquid two-phase microflows under the continuum hypothesis (Stone et al., 2003), certain types of forces control the two-phase flows in microfluidics as well as controlling droplet formation, for example, inertial force, viscous force, gravity, and capillary force. For relative comparison of these forces, several dimensionless numbers were introduced, including Reynolds (Re = ρvl/η), Capillary (Ca = ηv/γ), Weber (We = v2 ρl/γ), and Péclet (PeL = lvρCp/k) that depend upon the fluid-related parameters, the fluid's inherent characteristics, flow regime, and the geometric aspects. In addition, the viscosity ratio and flow rate ratio of the continuous phase to dispersed fluid are also utilized to characterize the behaviors of microfluidic flows (Maan et al., 2015; Muijlwijk et al., 2016).

#### 6.1.2 System geometries for droplet formation from microfluidics

Monodisperse drops may be produced by microfluidic devices of various geometries. The droplet generation mechanism relies heavily on the geometry of the microfluidic device. Based on the types of driving force for drop generation, the latter may be grouped into shear-induced geometries, interfacial tension-induced geometries, and external force-induced geometries. Capillary assemblies and planar geometries belong to shear force-driven geometries. A capillary assembly may be co-flowing or flow-focusing, and a planar device typically consists of linked microchannels with a rectangular cross-section to ensure the fluid flows within the same plane (Ushikubo et al., 2014). Surface tension-driven geometries imply that droplet formation is surface tension-driven, which is achieved through a stepped geometry. The size and shape of the droplet in this device have a close correlation with the height of the step. Discoid droplets will be produced when the step is shallow, and more desirable spherical droplets will be produced when the step is deep (Kobayashi et al., 2006). Based on the idea suggested by Nakajima et al., this surface tension-induced method was widely known as “microchannel emulsification” (Kawakatsu et al., 1997). This strategy is usually classified into two types based on the type of microchannel (grooved or straight-through). External force-driven geometries deal with employing an external force as the driving force, which propels the microfluid locally and responds rapidly to prepare the droplets, and thus droplets are created passively. External forces used for that purpose can be electric, magnetic, thermal, mechanical, etc. (Shang et al., 2017).

### 6.2 Application of microfluidics in different types of food fraud

#### 6.2.1 Detection of adulterants in liquids (oils, beverages, etc.)

Microfluidic platforms have been investigated for rapid detection of adulteration in edible oils. For example, a paper-based microfluidic device was reported for detecting palm oil adulteration in sunflower oil, with discrimination reported over the 10–50% (*v*/v) range (Muthukumar et al., 2020).

#### 6.2.2 Allergen testing and cross-contamination detection

Microfluidic methods improve the capability of detecting food allergens by integrating sample handling and analytical detection into miniaturized platforms.

Microfluidic methods immensely improve the capability of detecting food allergens promptly and sensitively. They permit assay time reduction from hours to 15–20 min or even less, compared with conventional ELISA approaches that could take hours. The microfluidic biosensor can offer food safety inspectors and industries the advantage of choosing assay protocols for precisely targeted allergens, making it a highly multifunctional platform for ELISA applications (Weng et al., 2016). In addition to identifying better biomarkers for the diagnosis of food allergy, it should also be possible to detect the biomarkers in a rapid, non-invasive, and reliable manner. This is best served by microfluidics. The most advanced is the microfluidic use of sIgE tests, of which many already have commercial forms. Some are point-of-care compatible with rapid diagnosis, having a time to result between 5 min and tens of minutes (Castaño et al., 2020).

Most microfluidic platforms for the diagnosis of food allergy have focused on multiplexing and miniaturizing sIgE tests for multiple allergens. The methods perform immunocapture of sIgE onto allergen-functionalized channel surfaces or particles (Castaño et al., 2020).

#### 6.2.3 Pathogen detection: microbial safety in food products

Microfluidic methods of pathogen detection using nucleic acid amplification can provide rapid and sensitive analysis within miniaturized platforms.

**Microfluidic methods of pathogen detection.**

(a) ***Nucleic Acid Amplification***

Microfluidic methods of pathogen detection using nucleic acid amplification have transformed food safety analysis. Microfluidic methods allow for speedy and sensitive amplification of particular DNA or RNA sequences from diluted samples, making it possible to detect foodborne pathogens including bacteria, viruses, and fungi. Microfluidic devices integrating the preparation, amplification, and detection steps improve efficiency and specificity with less analysis time and reagent consumption. For example, microfluidic PCR devices can identify pathogens such as Salmonella and *E. coli* within less than an hour and at lower concentrations, thus enhancing food safety protocols considerably (Kasputis et al., 2024).

The key advantages of microfluidics are seen in portability, ease of use, and the ability to combine sample preparation, nucleic acid amplification, and readout processes seamlessly. The range of material compositions offers various advantages (Kasputis et al., 2024).

(b) ***Immunoassay***

Immunoassays are a general screening technique for foodborne pathogens. Immunoassays employ antibodies to detect specific antigens present in pathogens and are thus applied in food safety analysis. Immunoassays can be effectively integrated into microfluidic devices. Integration of immunoassays into microfluidic devices permits rapid and effective testing, and this is important for quick food safety analysis. Miniaturization of such assays in microfluidic systems renders them more portable and user-friendly, particularly for low-resource settings: Immunoassays are in a position to produce quick results, which is crucial for site-specific testing. Immunoassays are extremely sensitive and can identify minimal traces of pathogens in food samples.

#### 6.2.4 Identifying DNA and protein signatures for species authentication

Microfluidic platforms can miniaturize DNA- and protein-based authentication workflows, allowing sample processing, amplification, and detection to be integrated into compact devices. This approach has been investigated particularly for rapid species identification and meat adulteration screening. Microfluidic devices, for example, PCR-driven microfluidic lateral flow strip devices, can detect numerous species of meat products simultaneously with detection limits as low as 0.01% adulteration (Shi et al., 2023).

A new microarray chip PCR-guided microfluidic lateral flow strip (LFS) device has been invented for accurate and efficient detection of meat adulteration in beef processed food. The method applies four primer sets for target species-specific genes for the detection of chicken, duck, and pork. The novel platform facilitates the direct deposition of dsDNA amplicons onto the LFS without pipetting, minimizing the likelihood of carryover contamination—a historical phenomenon for conventional PCR-LFS platforms.

Compared to traditional qPCR methods that cost approximately 100–200 RMB for four samples, the microfluidic LFS device offers an affordable alternative of less than 20 RMB per four samples, which is more suitable for large-scale screening. With the integration of sample loading, detection, and reporting into a single workflow, this technology allows a simple, high-throughput method of detecting meat fraud and ensuring food safety (Wang et al., 2023).

#### 6.2.5 Analyzing emulsifying and rheological properties for quality control

Microfluidics allows the emulsification to be controlled, which is vital in food product evaluation, for example, of dressings and sauces. Techniques such as shear-driven and spontaneous emulsification enhance emulsion quality and stability. Microfluidic devices can quantify the flow and deformation of food materials, providing data on texture and consistency, which are significant in quality control (Qiao et al., 2023).

In recent years, the characterization of rheological and emulsifying properties has played increasingly significant roles in ensuring quality control in structuring food processes with the support of microfluidics. Emulsifying properties, which are responsible for food emulsion stability and acceptability, require parameters like long-term stability, distribution, and particle size to be quantified (Abate & Weitz, 2009). Moreover, rheological properties, such as viscosity and elasticity, play a key role in the understanding of food material flow behavior when stressed and significantly influence processing behavior and sensory attributes (Maan et al., 2015). By intensive research of such properties, food technologists can ensure the required texture, stability, and mouthfeel of food products, thus resulting in consumer acceptability (Comunian et al., 2017). Since microfluidic approaches are being proposed, these parameters can be manipulated with more control to create good-quality foods having desired characteristics (Zhang, Xu, et al., 2020). To facilitate optimization scenarios of production as well as maintenance of food line uniformity, continuous monitoring of rheological as well as emulsifying characteristics is crucial.

Across these applications, the reviewed studies demonstrate that microfluidic platforms can be adapted to different food-fraud targets, including chemical adulterants, allergens, pathogens, and species-specific markers. However, the analytical requirements differ considerably among these applications. Chemical adulterants may require highly sensitive chemical or electrochemical detection, whereas species authentication generally depends on nucleic-acid or protein-based recognition. Therefore, the effectiveness of a microfluidic platform should be evaluated according to the target analyte and food matrix rather than assuming a single platform can address all forms of food fraud.

### 6.3 Advantages of microfluidics for low-resource settings

These microfluidic technologies provide low-cost, rapid, and highly sensitive analytical solutions, which are especially useful in the case of food fraud detection in low-resource settings. Microfluidics, by its central position in Lab-on-a-chip (LOC) technologies, is crucial to the design of rapid, quantitative, and sensitive diagnostic devices. The technology can reduce costs significantly by cutting down on both analysis time and reagent usage. Presently, the most interesting use of LOC technologies is in early and precise diagnosis of infectious diseases in resource-constrained developing countries. Microfluidic R&D, however, tends to be confined to those countries with abundant research resources because available prototyping techniques for microfluidic devices are expensive and the availability of materials is scarce in low-resource environments (H. Nguyen et al., 2018). Microfluidic devices, being different from classical laboratory-based solutions, are conducive to smartphone-based detection or paper-based sensors and can offer field-level food verification without the assistance of a complicated infrastructure or specialist expert (Ray et al., 2022). This is particularly useful in food fraud prevention in resource-scarce environments (Yetisen et al., 2013).

(1) ***Cost-Effectiveness***: Since paper-based microfluidic chips are inexpensive to produce, they can be employed in low-resource environments. Due to their low-cost nature, they can be applied extensively in conditions where standard lab equipment is not viable (Liang et al., 2023).
(2) ***Quick Detection***– It allows for quick analyses and conclusions, which is essential in speedy food safety screening. Rapid detection systems may prevent foodborne illness by enabling immediate response  (Liang et al., 2023).
(3) ***Reduced Usage***: Microfluidic-based paper systems use low quantities of reagents and samples, which is ideal when resources are minimal. To promote sustainability and mitigate financial burden, low consumption is appropriate (Liang et al., 2023).
(4) ***Multi-analyte Integration***: Microfluidic systems can be integrated with various detection methods, such as colorimetric and fluorescence analysis, which is very convenient for food safety applications. Thus, more tests can be performed on the same platform (Liang et al., 2023).
(5) ***Field Testing:*** The one great advantage is that test can be done at any place. There is no requirement for fancy labs to do it. It's really useful where there are no labs to access (Liang et al., 2023).
(6) ***High Sensitivity:*** These sensors can detect trace amounts of contaminants. This is especially crucial in maintaining food safety. They are able to identify even minute amounts of harmful substances (Jiang et al., 2024).
(7) ***Simple to Operate:*** Microfluidic systems are simple to automate. That is, there is no requirement for a large number of skilled laborers to operate complex lab equipment. It's advantageous for institutions that lack trained staff (Jiang et al., 2024).
(8) ***Field-Relevant Application***: Microfluidic technology is portable and small in size. This means that it can be moved from one place to another, even to remote areas. Portability enables the ability to carry out testing in the field, which is essential in rapid track evaluation of food safety without any complicated laboratory setup (Trinh et al., 2023).
(9) ***Portable***: Microfluidic technology is portable and small in size. This means that it can be moved from one place to another, even to remote areas. Portability enables the ability to carry out testing in the field, which is essential in rapid track evaluation of food safety without any complicated laboratory setup (Trinh et al., 2023).
(10) ***Reagent and Sample Cost Savings:*** Microfluidic technology is portable and small in size. This means that they can be moved from one place to another, even to remote areas. Portability enables the ability to carry out testing in the field, which is essentialforn rapid track evaluation of food safety without any complicated laboratorysetupp (Trinh et al., 2023).
(11) ***Multiplexing of Multiple Tests:*** Microfluidics may be designed to incorporate multiple detection technologies into one chip, thus facilitating thorough testing of numerous contaminants in a single test. This attribute is advantageous in limited resource settings where thorough testing of foods should be done, although it may be rather cumbersome (Trinh et al., 2023).

Taken together, these advantages indicate that microfluidics is particularly suited to screening and preliminary authentication where rapid decision-making is required. Nevertheless, portability and low sample volume should not be considered substitutes for analytical validation. For practical adoption, sensitivity, selectivity, reproducibility, matrix tolerance, and operator-independent interpretation must be demonstrated alongside portability and low cost.

#### 6.3.1 Lab-on-a-Chip (LOC) devices

Lab-on-a-chip technology miniaturizes all laboratory operations into a single chip for enhanced portability and automation (Whitesides, 2006b).

Recent advancements comprise the application of artificial intelligence (AI) and machine learning in real-time analysis of data that enhances sensitivity and accuracy for the detection of fraudulent foods. Microfluidic PCR and immunoassays have been incorporated into LOC systems to enable better detection of pathogens and contaminants in field-deployable and on-site applications (Nguyen et al., 2019).

#### 6.3.2 Microfluidic electrochemical sensors

Microfluidic electrochemical sensors offer high selectivity, sensitivity, and in situ monitoring capabilities (Nguyen et al., 2019). Microfluidic electrochemical sensors have been widely used for the detection of heavy metal impurities in seafood, food additives of synthetic dyes, and artificial sweeteners in processed foods.

#### 6.3.3 Smartphone-integrated microfluidic systems

The combination of microfluidics and smartphones provides real-time analysis and data acquisition, improving user convenience and access to food fraud detection (Xu et al., 2020). Microfluidic-based smartphone systems have been employed for the successful analysis of food colorants, verification of organic food labeling, and the detection of food fraud. Smartphone integration can support image-based readout, portable data acquisition, and remote recording of test results, thereby facilitating field-level food analysis.

## 7 Case study: real-world examples of food fraud detection and prevention

The following case studies provide study-specific evidence of the analytical performance and practical application of food-fraud detection technologies, with emphasis on reported detection limits, accuracy, response time, and portability**.**

### 7.1 Milk adulteration detection

In many developing countries, including India, Pakistan, and China, the addition of adulterants such as urea, detergent, and hydrogen peroxide to milk is a serious health concern. As a solution to this challenge, Subhashis Patari et al. (2022) designed a novel three-dimensional paper-based microfluidic system that can detect seven different adulterants with fast color changes in 1–2 mL of milk withina few minutes. This portable system has a detection limit of 0.05%–0.2% with a recovery percentage of 85%–107%. In comparison to other techniques like HPLC and mass spectrometry, the cost-effective nature of this microdevice makes it an ideal choice for detection. It took less than 30 s to provide test results than other laboratory methods.

Additional advancements in milk authentication include:

• **PCR-based methods** detecting buffalo, goat, and sheep milk mixed with cow milk (Giglioti et al., 2022).
• **Laser-Induced Breakdown Spectroscopy (LIBS)** identifying bovine milk adulteration with caprine and ovine milk (Sezer et al., 2018).
• **Smartphone-integrated digital colorimetry** identifying water dilution in milk (Silva & Rocha, 2020).

### 7.2 Detection of aflatoxin M1 and other contaminants

A portable and cost-effective paper-based microfluidic device (μPAD), which uses AuNPs, has been designed by Kasoju et al. (2020) for the detection of Aflatoxin M1, a milk contaminant associated with liver damage and cancer. While techniques such as HPLC and ELISA are quite costly and require a lot of time, this μPAD changes its color from red to blue upon detecting Aflatoxin M1. This μPAD has a detection limit of 3 pM in water and 10 nM in milk, lasts up to three months, and is best suited for field tests.

Likewise, Selvaraj and Ravi Shankaran (2023) developed a nano-enabled plasmonic μPAD for melamine detection, with the change in color from orange-red to purple indicating contamination at a 5.0 × 10^−5^ M detection limit. Moreover, Ray, Prabhu, Prasad, Garlapati, et al. (2022) and Ray, Prabhu, Prasad, kumar Garlapati, et al. (2022) developed a wax-printed μPAD for starch adulteration by using the iodine-starch colorimetric reaction, and its detection limit is 0.05%.

The problem of milk fraud, such as misleading labeling of UHT milk as fresh and manipulation of fat content, is becoming more prevalent in the dairy industry. In this study, Field Flow Fractionation (FFF) along with chemometrics was employed for the analysis of 47 milk samples, allowing differentiation between UHT and non-UHT milk due to the detection of heat-induced changes in protein aggregation. This technique allowed tracing milk back to the manufacturing plant, which in turn demonstrated that various milk brands usually come from the same production site. Moreover, milk was classified based on fat content (whole, semi-skimmed, skimmed). This technique proved to be superior to the conventional Gerber method of fat content estimation. As compared to common HPLC and NMR techniques, this technique does not require any organic solvents and allows analysis of samples in less than an hour (Giordani et al., 2024).

### 7.3 Honey authentication using NIR spectroscopy

Honey adulteration, which involves mixing glucose syrup with honey, can be easily detected by the use of a sensitive amperometric biosensor that is coupled to the screen-printed carbon electrode (SPCE). In spite of being very sensitive, stability of the biosensor is only achieved for five days, making the biosensor appropriate for immediate analysis (Julika et al., 2020).

Biswas & Chaudhari, 2024 note that NIR spectroscopy is the most appropriate method to detect honey adulterants such as high-fructose corn syrup (HFCS), where 98.7% accuracy can be achieved (Bázár et al., 2016). The use of machine learning approaches, such as SVM and ANN, further improves accuracy (Benković et al., 2022).

### 7.4 Microfluidic detection in food fraud prevention

#### 7.4.1 Egg adulteration

Adulteration of EGGs: A novel technique for detection of tea-coated broiler eggs passed off as country eggs is introduced by Ray et al. (2023a, 2023b). The test makes use of paper-based microfluidic devices wherein ferric chloride (FeCl₃) is used to develop a dark blue precipitate with tea polyphenols, while citric acid is used to remove the stain and reveal the white color of the eggshell. An automated AI-based application named “Eggo” can detect these with 100% accuracy within five minutes. The chemical reactions have been validated by FTIR spectroscopy; out of six field samples tested in Karnataka, only one was found to be a fake one.

#### 7.4.2 Essential oil adulteration

Detection of essential oils: Essential oils (EOs) are frequently used in food, cosmetics, and medical applications. However, the volatile nature and demands of the market make them prone to adulteration. The review by Yang et al. (2024) examines the use of sensory evaluation, physicochemical characteristics, and chemical composition analysis as crucial methods of authentication. The sensory evaluation, which involves both a human sensory panel and technology-based sensors, distinguishes between authentic and adulterated products by using odor and texture properties. The physicochemical analysis involves the evaluation of density, refractive index, optical rotation, and acid and ester value, with any difference indicating the presence of potential adulteration. Chemical analysis identifies significant compounds such as terpenes and aldehydes and changes due to oxidation and fraudulent activities.

#### 7.4.3 Cooking oil adulteration

The adulteration of cooking oil, in particular the mixing of cheap oils such as palm oil with premium oils like sunflower oil, was successfully detected using μPAD assays. The detection of palm oil in sunflower oil was done using μPAD analysis, which involved analysis of the greyscale intensity of the sample, revealing a linear relationship within the 10–50% level (Muthukumar et al., 2021). Adulteration of cow ghee with both palm oil and sunflower oil was conducted through the μPAD that was impregnated with 2,2-Diphenyl-1-picrylhydrazyl (DPPH) as a color indicator, detecting 2.5% palm oil and 1% sunflower oil levels (Naik et al., 2023).

This case serves to emphasize an important benefit of using μPAD technology: the possibility of transforming the relatively complicated task of adulteration into a fast screening method. Nevertheless, it would be important to test the applicability of these tests for different types of oil, different types of adulterants, and different concentrations because food matrices might have an impact on assay reaction.

The case studies presented above demonstrate a common trend toward moving from laboratory detection to portable testing. Nevertheless, the performance of each particular device is usually evaluated on the basis of certain experimental conditions and food matrices. Therefore, the main challenge now is not limited to the development of sensitive microfluidic methods anymore. It is necessary to validate these tests.

### 7.5 Functional food and dairy fraud detection

#### 7.5.1 Functional food adulteration

The Nanjing Institute for Food and Drug Control and the National Institute of Metrology in China conducted a study on illicit pharmaceutical additives in functional foods using the MRSIT-HRMS (Multiple Sample Rapid Introduction combined with High Resolution Mass Spectrometry) technology, a rapid screening method that does not require chromatography. Researchers tested 50 batches from the Nanjing Municipal Public Security Bureau and discovered 41 samples that were heavily contaminated with sildenafil, tadalafil, sibutramine, ephedrine, and furosemide, all of which are used to treat erectile dysfunction, lose weight, and regulate blood pressure. The technique achieved 100% accuracy, which is comparable to the reliability of LC-MS/MS (Hu, Zou, et al., 2024b).

#### 7.5.2 Parmigiano Reggiano cheese authentication

Traditional authentication techniques, such as chemical tests and NIR spectroscopy, struggle to detect quality differences in Parmigiano Reggiano cheese. Becchi et al. (2024a, 2024b) utilized untargeted metabolomics (UHPLC-MS) and machine learning to identify markers linked to ripening, altitude, and rind inclusion. The model achieved over 60% accuracy in detecting excessive rind content (>18%).

#### 7.5.3 Pomegranate juice and wine adulteration

Pomegranate juice adulteration: Hu and Lu (2020a) at the University of British Columbia in Canada developed a low-cost, portable Lab-on-a-Chip that detects pomegranate juice contamination by cheaper juices such as apple and grape. Fresh juices were stored at −20 °C, while counterfeit samples were produced in 1:1 and 1:1:1 ratios. Two DNA extraction methods were evaluated: the Qiagen DNeasy Kit and the Fusion 5 paper-based method. LAMP primers were used to target species-specific DNA (matK for grape, ITS for apple and pomegranate) at 63 °C for 45 min. The LOC device included seven layers and contained a Fusion 5 DNA extraction zone, microfluidic flow channels, and a portable heater. A fluorometric dye system detected positive samples (green under UV light) and negative samples (black), and the results were recorded on an iPhone 6.

The gadget detected pomegranate DNA at 10 pg and grape/apple DNA at 100 pg, with a limit of detection (LOD) of 2 μL for pomegranate and 5 μL for apple/grape. The authentication process was speedy (∼1 h), reliable, and field-ready, with a cost of $4 per test. The study established LOC-LAMP technology as a viable approach for detecting food theft, benefiting regulators, manufacturers, and inspectors. Future improvements include larger DNA databases and smartphone-based result interpretation**.**

Wine adulteration: Smartphone-based digital image analysis is a more cost-effective and practical way to examine the authenticity of Slovak Tokaj wine compared to traditional methods. Researchers evaluated 63 wine samples (41 from Slovakia and 22 from other locations) with a Samsung Galaxy S8 in a customised cubic cage with controlled luminance. MATLAB was used to process photos and generate histograms in greyscale, RGB, and HSV. Three chemometric models were investigated, including OC-PLS, DD-SIMCA, and PLS-DM. DD-SIMCA and PLS-DM reached 100% accuracy; however, OC-PLS had lower specificity (65%). Chemometric modelling using smartphone pictures is a cost-effective and efficient way to authenticate wine and prevent fraud (Onça et al., 2024).

The pomegranate juice study illustrates how molecular amplification can be integrated with a portable microfluidic platform to achieve species-specific authentication outside conventional laboratory settings. At the same time, the requirement for target-specific primers indicates that such systems are better suited to predefined fraud scenarios than to broad, non-targeted screening. Expansion of target databases and multiplexed assays could therefore improve their applicability to more complex adulteration patterns.

#### 7.5.4 Alcoholic beverage adulteration

Whisky dilution and caramel addition can be identified using redox titration-based μPADs (Rezende et al., 2022). More concerning is the fraudulent addition of paracetamol (PAR) in whisky. Moreira et al. (2024) designed a distance-based μPAD that detects PAR through a Prussian blue reaction, with a detection range of 15–120 mg/L.

### 7.6 Tuna and meat adulteration detection

#### 7.6.1 Tuna species authentication

Tuna fish: Food fraud in the tuna sector is a major concern due to the economic worth and price disparities across species, which lead to mislabelling and substitution. Traditional morphological identification is useless for processed tuna, while DNA-based approaches are prone to degradation. This work used metabolomics-based mass spectrometry to distinguish between skipjack tuna (*Katsuwonus pelamis*), bigeye tuna (*Thunnus obesus*), and yellowfin tuna (*Thunnus albacares*) utilising UPLC-Q/Orbitrap MS for non-volatile metabolites and GC-Q-TOFMS for volatile metabolites. Tuna samples from the Indian Ocean were held at −60 °C, crushed in liquid nitrogen, and extracted with a mixture of acetonitrile, methanol, and water (2:2:1, *v*/v/v). A total of 77 metabolites (UPLC-MS) and 22 metabolites (GC–MS) were discovered, with 38 biomarkers differentiated using PCA and OPLS-DA, and verified using Hierarchical Cluster Analysis (HCA) and heat maps. Bigeye and yellowfin tuna displayed comparable profiles; however, skipjack tuna had different amino acid and lipid compositions. KEGG pathway enrichment revealed 27 metabolic pathways, such as glutamine metabolism, phenylalanine biosynthesis, and fatty acid metabolism. This work indicates that metabolomics-based authentication is a useful technique for detecting tuna fraud, providing regulatory bodies, seafood traders, and consumers with accurate species discrimination. (Hu, Zhu, et al., 2024).

#### 7.6.2 Meat adulteration in halal foods

Meat adulteration, particularly the mislabeling of pork as halal meat, was identified using a hybrid μPAD combined with solid-phase extraction. DNA fragments were selectively captured using polydopamine-modified magnetic nanoparticles and analyzed via colorimetric detection, achieving a detection limit of 4.59 nmol/mL. This method provides a reliable solution for ensuring halal food authenticity and preventing religious and ethical fraud.

## 8 Challenges and limitations in current food fraud detection practices

Advances in food fraud detection have resulted in tremendous gains, but numerous hurdles remain, impeding their effectiveness and widespread use. These limits are due to the complexity of food matrices, technological constraints, regulatory gaps, and hurdles to industrial integration. Addressing these problems is critical for improving detection accuracy, assuring regulatory compliance, and facilitating real-world application.

1. Matrix complexity and interference issues: Many detection techniques are challenging to use because food matrices are complex and contain a variety of physical and chemical components that can interfere with analytical results. Microfluidic paper-based analytical devices (μPADs) have limited sensitivity and specificity for identifying adulterants in dairy, honey, and culinary oils due to matrix effects.
2. Limited multi-analyte detection capabilities: While classic techniques such as HPLC and GC–MS can detect many pollutants in a single test, many microfluidic and biosensor-based approaches are only designed to detect a single analyte. This limits their application for comprehensive screening of numerous adulterants concurrently (Vinothkanna et al., 2024).
3. Lack of standardization and regulatory barriers: The lack of global standardization for fast detection technologies impedes their use in regulatory enforcement. Studies on functional food adulteration reveal variations in the validation of novel detection systems, affecting global adoption (Hu, Zou, et al., 2024b).
4. Sensitivity and Detection Limits: Rapid detection technologies, such as μPADs and electrochemical biosensors, still struggle to reach laboratory-level detection limits. In essential oil authentication, colorimetric tests exhibit larger detection limits than chromatography-based techniques, limiting their usefulness in identifying slight adulteration (Yang et al., 2024).
5. Integration with digital platforms for data interpretation: While smartphone-based detection is convenient, variations in camera resolution, lighting conditions, and human interaction might cause inconsistencies. A study on egg adulteration detection discovered that smartphone photography performance varied greatly depending on the environmental circumstances (Ray et al., 2023b)
6. Cost and accessibility concerns: Despite being low-cost alternatives, some microfluidic devices and portable biosensors necessitate specialized reagents and production procedures that may not be readily available in all areas. According to research on pomegranate juice authentication, pricing and accessibility continue to be major barriers to wider use.
7. Industrial-Scale Implementation Challenges: Many detection systems are still in the research stage and lack scalability for real-time industrial applications. For example, research on heavy metal pollution detection utilising microfluidic platforms shows that, while promising, these systems require more improvement before mass deployment (Guan & Sun, 2020a, 2020b).
8. Cross-reactivity and false positives:
  Biosensor-based methodologies, such as aptamer-based and immunoassay assays, might demonstrate cross-reactivity, resulting in false-positive results. In meat authenticity investigations, molecular assays occasionally misidentified species due to protein cross-reactivity (Becchi et al., 2024b)

The review of the existing literature shows that the main challenge for the implementation of this concept does not lie in the lack of ideas to detect food fraud, but rather in the lack of transition from concept testing to the development of validated methods. Further research needs to focus more on practical aspects such as reproducibility and shelf-life.

**Future Directions to Overcome These Challenges:**

**Improving Multi-Analyte Detection:** Advances in lab-on-a-chip technology may allow for the simultaneous detection of several adulterants, improving real-time monitoring efficiency.

**Standardization and Validation:** Regulatory organizations should collaborate to develop harmonized rules for rapid detection instruments to guarantee consistent and trustworthy results.

**Enhancing Sensitivity Using Nanotechnology:** The use of nanomaterials in biosensors can reduce detection limits, making rapid tests more competitive with traditional approaches.

**AI-Driven Analysis and Automation:** Machine learning models may improve pattern recognition and interpretation of results, potentially decreasing human error in digital detection systems.

**Industry Adoption and Cost-Effective Scaling:** Increased collaboration among researchers, food manufacturers, and regulatory bodies has the potential to hasten commercialization and reduce costs.

## 9 Conclusion and future perspectives

Nevertheless, traditional analytic methods such as chromatography, spectroscopy, and DNA analysis are highly valuable for reliable detection of fraud in food. Nonetheless, due to the need for special equipment, skilled personnel, and laboratory conditions, they are not always suitable for rapid on-site detection of food fraud. From the literature review conducted, one can conclude that the value of microfluidics in food fraud detection is based on the possibility of the integration of sample preparation and analysis into a compact, portable, and relatively cheap device. Microfluidic technologies are applied in such areas as adulterant detection, allergen screening, pathogen detection, and food authentication.

The reviewed literature provides sufficient information to make conclusion that the main advantage of microfluidic systems does not consist in replacement of laboratory techniques but in the combination with these methods and making rapid screening decisions possible in the places without conventional laboratories. Nevertheless, many applications have been proved only under experimental conditions in the case of specific matrices. Thus, future researches should focus on increasing sensitivity, selectivity, decreasing cross-reactivity, overcoming matrix effects, and proving reproducibility on diverse real-life samples. Multiplexed systems, which can detect several indicators of fraud simultaneously, may become a new trend in this field.

For implementation from an industrial point of view, issues like scalability, cost-effective manufacturing, stability of devices, testing processes, and performance of analysis should be considered. Cooperation between researchers, food industries, regulatory organizations, and technology developers will be crucial for setting up proper validation and regulation frameworks for portable food fraud detection devices.

Combining the use of microfluidics with smartphone technology, artificial intelligence, and digital traceability could help achieve rapid on-site food fraud detection and analysis. Nevertheless, the successful implementation will be based on analysis validation, reproducibility, testing protocols, regulations, and performance on actual food samples. In general, microfluidics is a promising technology for rapid and decentralized food fraud screening.

## Authors contribution

POC, MKG, ADT, AA, AH conceptualized, wrote and edited the manuscript. ADT, AA, AH supervised the work. MA, VB, DS, NP, HKR edited the manuscript.

## CRediT authorship contribution statement

**Pratikshya Oli Chhetri:** Writing – review & editing, Writing – original draft, Conceptualization. **Muskan Kumari Gupta:** Writing – review & editing, Writing – original draft, Conceptualization. **Mradul Awasthi:** Writing – review & editing, Software. **Vinay Balodi:** Writing – review & editing, Software. **Ankita Hooda:** Writing – review & editing, Supervision. **S. Dhivya:** Writing – review & editing, Software. **Nikhil Pancholy:** Writing – review & editing, Software. **Himanshu Kumar Rai:** Writing – review & editing. **Abhishek Dutt Tripathi:** Writing – review & editing, Supervision, Conceptualization. **Aparna Agarwal:** Writing – review & editing, Supervision, Conceptualization.

## Funding

No funding received.

## Declaration of competing interest

The authors declare that they have no known competing financial interests or personal relationships that could have appeared to influence the work reported in this paper.

## Data Availability

Data will be made available on request.
